# Cases Illustrative of the Effects of Iodine

**Published:** 1828-06

**Authors:** Jeremiah H. Brower

**Affiliations:** Elizabethtown, Ohio


WESTERN JOURNAL

OF THE

MEDICAL & PHYSICAL SCIENCES.

JUNE, 1828.

### ESSAYS AND CASES.

---

### Art. I.—*Cases illustrative of the Effects of* Iodine. By Jeremiah H. Brower, *of Elizabethtown, Ohio.*

Case 1. N. M., aged 19, of light complexion and strongly marked scrofulous habit, about six months since, perceived a small tumour, on the side of the neck under the lobe of the left ear, which gradually increased in size, though unattended with pain. When she applied to me for assistance, a cluster of enlarged glands, about the size of an egg, extended from the angle of the lower jaw to the sternal end of the clavicle, hard and unyielding in consistence, and very irregular and lobulated in structure. Her general health had been much impaired for some time previous, being constantly harrassed with a dry, hard cough, without any expectoration. She complained of a sense of straitness and difficulty of breathing, and was much emaciated: her extremities were somewhat œdematous: digestion perfect, and catamenia regular. From these circumstances, connected with the fact of two members of her family having recently died of confirmed phthisis, I was induced to believe, that a tubercular affection of the lungs was in progress. The ordinary routine of treatment for similar local affections, was diligently pursued for some time, with no apparent effect. I then resolved to prove the effect of the Iodine, and accordingly prescribed the tincture, made according to the following formula:—℞. Hydriodat. Potas: ℈ij. solv. in sp: Rectif. ℥v; 15 drops to be taken in a little mint water, 3 times a day: she was also ordered to rub the tumours well once, twice or thrice a day, with the following liniment: ℞. Tinct. Iodin. ℈ij, Lin. Sæpon. ℥ij. mix. On the sixth day of this treatment, a perceptible diminution of the tumour had taken place; and the skin over it, which had been tense and adherent to the parts, could be readily corrugated: her cough had greatly diminished, and her breathing was less difficult and laborious. As her pulse was now full and bounding, and she complained of severe headache, she was bled to 18 oz. and directed to omit the tincture for two days. She then resumed it, and gradually increased the dose to 25 drops, thrice a day: the tumours meanwhile rapidly decreasing, and with them the cough and pulmonic irritation. At the expiration of six weeks, the swelling had disappeared, and her general health was almost re-established; her strength and flesh were rapidly recruiting; and every symptom of incipient phthisis had vanished. This case occurred in the month of February. I saw the patient a few days since, she continues free from complaint, and has become comparatively fleshy.

Case 2. John C., a stout healthy lad of 18, was, in the summer of last year, thrown in wrestling, the knee of his antagonist striking and violently bruizing the scrotum and testes: he immediately fainted from the exquiste pain, and, about six weeks afterwards, perceived the right testis considerably enlarged, and, occasionally, after hard work, to be extremely painful. It continued to increase rapidly, until, being no longer able to conceal it from his parents, a respectable surgeon was consulted, who pronounced it a scirrhus of the testis, and proposed immediate extirpation as the only safe treatment. The patient, not relishing this advice, applied to me for assistance. The right testis was now enlarged to the size of a goose egg; hard and uneven on its surface, and very little painful on pressure. The skin was natural and healthy in its appearance, and adherent to the testis in several places. The epidydymis was enlarged, and its convolutions painful: the cord, also, was diseased as high up as it could be felt. This circumstance seemed to contra-indicate an operation. Upon its anterior surface were two or three superficial abscesses, which, when opened, discharged a small quantity of thin pus. For several weeks previous, he had, frequently, acute pains darting up the back and loins; and, besides the inconvenience felt from his back, his general health had become considerably impaired. Under the impression that the evident derangement of the digestive function might have some influence on the local disease, he was ordered to take Blue Pill and infus. Quassiæ; and to rub the tumour twice a day with camphorated mercurial ointment. By this treatment the general health was restored, the mouth having been slightly affected; but, at the expiration of a month, no diminution of the bulk or pain of the testis was perceptible. A variety of discutient applications were then used; and, for two weeks, the electrical shock was passed through it. I then, as a last resort, directed him to take 20 drops of the Tinct. Iodine twice a day, and to rub the part well with the liniment: the scrotum was suspended by proper bandages, and absolute rest enjoined. On the fourth day after commencing this treatment, the soreness of the tumour and darting pains left him, and he complained of a prickling sensation in it. He was obliged to omit the tincture for two or three days, as it produced distressing head ache and nausea, which were relieved by bleeding. By this treatment, at the expiration of four weeks, during which period he was obliged to lay aside his medicines several times, the testis was diminished to the size of a hen's egg; the skin of the scrotum became flaccid and corrugated as usual; and, at this time, the patient remains free from complaint, and no difference can be perceived in the size of the testes.

Case 3. J. Roberts, aged 56, was attacked in September last, with (as I suppose) general inflammatory rheumatism, which seated itself more particularly in the shoulders, back and loins. By active depletion, &c. his fever and pains left him; but, as he convalesced, he perceived a remarkable degree of weakness in the small of the back, with a dull heavy pain, and, occasionally, a difficulty in urinating; and sometimes there would be no alvine discharges for several days, and then he would have to resort to mechanical means to remove large quantities of hardened fæces. Supposing it to be the remains of his original disease, he paid but little attention to it, until he gradually lost all muscular power in his lower extremities, and became entirely unable to support himself or sit up. At this time (November) I saw him, and upon examination his general health was pretty good; appetite regular; a dull obtuse pain about the first lumbar vertebræ; his lower extremities perfectly paralysed, retaining their natural heat and sensibility, but the power of voluntary motion extinct, affording a fine specimen of Mr. Bell's distinction between the nerves of sensation and motion. To the ischuria, which at first troubled him, had succeeded a perpetual dribbling of urine,—manifesting a total suspension of the power of the sphincter vesicæ; and so entire was the loss of muscular energy in the rectum, that not a particle of fæces could be expelled without manual assistance. Upon a close examination of the vertebral column, no deviation from its regular and healthy aspect could be perceived, and percussion upon the spinous processes produced no pain or uneasiness; blisters, sinapisms, electricity and lotions, without number, had been applied; the Ung. Tart. Antim. had been used, liberally, without any effect. From the apparent integrity of the vertebral column, and the peculiar manner in which the disease was developed, I could not believe it to be a carious affection of the vertebræ or the intervertebral substance, so ably described and treated by Mr. Pott; and was convinced that the paralysis proceeded from a morbid thickening or enlargement of the theca vertebralis, lessening the calibre of the spinal canal, and thereby pressing upon the spinal cord, intercepting the nervous influence, and as forming a fair case for the trial of the Iodine. I accordingly ordered the tincture, 15 drops, three times a day, to be gradually increased, and a liniment composed of equal parts of Tinc. Iodin. Tinct. Lyttæ and Sp. Camph. to be rubbed in, from the neck to the sacrum. With a view to act upon the rectum and bladder, he took aloetic pills daily, and the Infus. Fol. Uv. Ursi, ad libitum. After pursuing this course for five or six days, excessive vomiting, head ache, and distressing sense of tightness across the breast, induced me to discontinue the medicines. He was bled from the arm to 18 oz. and 6 or 8 oz. more were abstracted by cupping on each side of the lumbar vertebræ; by which means he became relieved, and resumed the use of his medicines, which did not again produce any untoward symptoms, although he increased the dose of the Tinct. to 30 drops. In two weeks from this time, he could support himself upright in bed, and could void the fæces naturally, and retain his urine for two or three hours. From this period his recovery was unabated and rapid, and at the expiration of two months he was able to walk across the room without assistance, and the functions of the rectum and bladder were entirely restored.

The success of this interesting case afforded me the highest gratification, as illustrating, finely, the effects of this powerful agent, and justifying the diagnosis of the case.

Case 4. M. D., a sickly looking girl, aged 14, had, for two years, an indolent enlargement of the parotid gland, which gradually increased in size, until it had become as large as the segment of a hen's egg, producing great deformity and pain with difficulty in masticating. She was evidently scrofulous, and manifested strong indications of disease of the mesenteric glands. I directed 10 drops of the Tinc. Iodin. twice a day,—the iodine liniment to be rubbed in the tumour, and blue pill, with occasional laxative doses of rhubarb and magnesia. As the patient resided a considerable distance from me, I did not see her until three weeks had expired, when the enlarged gland had become reduced one half; her general appearance and health were greatly improved; appetite and digestion regular; her medicines had produced no unpleasant effect whatever; and she was ordered to increase the dose of iodine to 15 drops, and to continue her other medicines. In two weeks I saw her again: the tumour now had nearly disappeared, and every indication of returning health was manifested; she has become fleshy, and no vestige of the disease of the parotid remains.

I am now using the remedy in a case of incipient phthisis, in a decidedly scrofulous young woman, in which the past effects of the treatment give the most confident assurance of an entire cure being effected. In this case, also, the unpleasant influence of an over-dose of the article upon the sensorium and stomach, has obliged me to omit it for a few days, and employ venesection; which has uniformly relieved every unpleasant symptom.

It is not my purpose to enter into any disquisition as to the modus operandi of this important acquisition to the Materia Medica; but simply to communicate the results of my own experience with the article. Great discrepancy of opinion has existed with several eminent names, as to the utility and safety of the internal use of Iodine. Dr. Coindet, of Geneva, and Dr. Gairdner, discarding it as utterly unsafe and dangerous; while with others, of perhaps equal talent and discrimination, especially Dr. Manson, of Manchester, it has proved almost invariably safe and successful. "Quis sufficet tactas componere lites." Under these circumstances, it seems desirable to distinguish, accurately, the peculiar circumstances in which its use is indicated or may prove injurious; as it is only by patient and judicious investigation we can arrive at any fixed principles, on which to prescribe so powerful an agent upon the lymphatic system.

JEREMIAH H. BROWER.

*Elizabethtown, Ohio, April,* 1828.

